# Why conclusions from platinum model surfaces do not necessarily lead to enhanced nanoparticle catalysts for the oxygen reduction reaction†
†Electronic supplementary information (ESI) available: Data in Fig. 1 and 3, details of the electrochemical setup and measurements, method to assess the experimental *OH adsorption energies, theoretical details of the ORR modeling, assessment of free energies and generalized coordination numbers, schematics of reconstructed Pt(110) active sites on Pt_201_ and Pt_368_, zoom in Fig. 3's concave region. See DOI: 10.1039/c6sc04788b



**DOI:** 10.1039/c6sc04788b

**Published:** 2016-12-06

**Authors:** Federico Calle-Vallejo, Marcus D. Pohl, David Reinisch, David Loffreda, Philippe Sautet, Aliaksandr S. Bandarenka

**Affiliations:** a Leiden Institute of Chemistry , Leiden University , PO Box 9502 , 2300 RA Leiden , The Netherlands . Email: f.calle.vallejo@chem.leidenuniv.nl; b Physik-Department ECS , Technische Universität München , James-Franck-Str. 1 , D-85748 Garching , Germany . Email: bandarenka@ph.tum.de; c Univ Lyon , Ens de Lyon , CNRS , UMR 5182 , Université Claude Bernard Lyon 1 , Laboratoire de Chimie , F 69342 , Lyon , France; d Department of Chemical and Biomolecular Engineering , University of California , Los Angeles , CA 90095 , USA; e Nanosystems Initiative Munich (NIM) , Schellingstraße 4 , 80799 Munich , Germany

## Abstract

Experiments on model surfaces commonly help in identifying the structural sensitivity of catalytic reactions. Nevertheless, their conclusions do not frequently lead to devising superior “real-world” catalysts. For instance, this is true for single-crystal platinum electrodes and the oxygen reduction reaction (ORR), an important reaction for sustainable energy conversion. Pt(111) is substantially enhanced by steps, reaching a maximum at short terrace lengths of 3–4 atoms. Conversely, regular platinum nanoparticles with similar undercoordinated defects are less active than Pt(111) and their activity increases alongside the terrace-to-defect ratio. We show here that a model to design ORR active sites on extended surfaces can also be used to solve this apparent contradiction and provide accurate design rules for nanoparticles. Essentially, only surfaces and nanostructures with concave defects can surpass the activity of Pt(111), whereas convex defects are inactive. Importantly, only the latter are present in regular nanoparticles, which is why we design various concave nanoparticles with high activities.

## Abstract 2

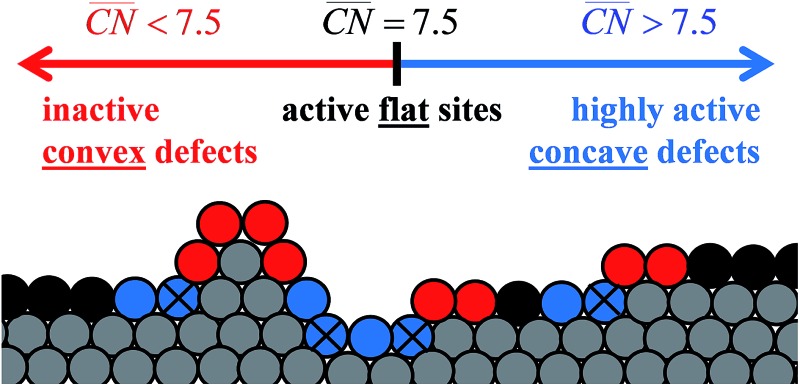
Platinum model-surface and nanoparticle catalysts for the oxygen reduction reaction are enhanced by the presence of concave sites.

## Introduction

Identifying active sites is one of the central paradigms in heterogeneous catalysis.^1–5^ However, this is a non-trivial task on solid catalysts due to the presence of multiple centers with dissimilar adsorption properties.^6,7^ The oxygen reduction reaction (ORR), which limits the efficiency of certain low-temperature fuel cells that will be important for the future use of renewable energy sources,^8–10^ is a prominent example of how complicated the process of finding active sites can be.^11^ For decades, a myriad of electrocatalytic materials have been tested for the ORR based on Pt and its alloys.^11,12^ Although Pt catalysts are known to be highly active materials, quantitative relationships between their geometric structure and activity are still missing. There is currently a gap between model surface and nanoparticle electrochemistry^13^ so that very often experiments on single-crystal electrodes result in design principles that do not lead to the elaboration of more efficient nanoparticle catalysts. At the same time, the high activity of certain nanostructures is in stark contrast with comparable single-crystal observations. This is true for numerous Pt-based catalysts, particularly stepped surfaces,^14–16^ nanoparticles of specific shapes^17–19^ and ordered arrays of them,^20^ mesostructured thin films,^21^ *etc.*

Volcano-type activity plots have shown that Pt(111) is the most active low-index surface of platinum.^11,12^ Its sites bind *OH, the archetypal ORR intermediate, slightly stronger (∼0.1 eV) than the optimal sites. It is known for late transition metals^22–24^ and in particular for Pt^25,26^ that highly coordinated sites bind chemisorbates (such as those involved in the ORR) more weakly than the undercoordinated ones. Following this idea, creating undercoordinated sites on Pt(111) should lower its ORR activity, which is the case on convex nanoparticles.^27,28^

However, multiple Pt catalysts with different types of undercoordinated sites^14–18,20,21,29^ demonstrate substantially higher activity than Pt(111). These opposing observations challenge our ability to understand activity trends using approaches based on adsorption energies only. Moreover, they reveal a lack of correlation between single-crystal and nanoparticle design principles that creates a gap between controlled laboratory experiments and technological implementations. In this work, we show that it is possible to determine the underlying features of a vast number of experimental data related to the high ORR activity of Pt stepped surfaces, nanoparticles of different shapes and arrays of them and other types of nanostructures using a single geometric descriptor. We will show that this descriptor, called generalized coordination number,^25,26,30^ enables a clear distinction between deleterious and beneficial defects for the ORR activity in nanoparticles as well as in extended surfaces.

## Methods

The DFT calculations of the stepped Pt surfaces were carried out with VASP,^31^ using the PBE functional^32^ and the projector augmented-wave method.^33^ The extended surfaces contained four metal layers, the two topmost of which as well as the adsorbates were allowed to relax in all directions, while the 2 bottommost layers were fixed at the bulk distances, with a Pt–Pt interatomic distance of 2.81 Å, typical of PBE. The relaxations were carried out with a plane-wave cut-off of 450 eV, using the conjugate-gradient minimization scheme, until the maximum force on any atom was below 0.05 eV Å^–1^. The *k*-point meshes (*k*_1_, *k*_2_, *k*_3_) were selected for extended surfaces so that their product with the norms of the lattice vectors (*a*_1_, *a*_2_, *a*_3_) in Å was (*a*_1_ *k*_1_, *a*_2_ *k*_2_, *a*_3_ *k*_3_) > (25 Å, 25 Å, 25 Å), which ensures convergence of the adsorption energies below 0.05 eV. The vacuum layer between repeated images was at least 14 Å and dipole corrections were applied. We used *k*_B_ *T* = 0.2 eV, and took the extrapolated total energies at 0 K. H_2_O and H_2_ were simulated in cubic boxes of 15 Å × 15 Å × 15 Å using the *Γ*-point and *k*_B_ *T* = 0.001 eV. We model the oxygen reduction reaction (ORR) following Nørskov *et al.*'s approach,^34^ which is explained in detail in Section S2 in the ESI.†

A comprehensive picture of Pt ORR catalysts can only be provided using a descriptor able to capture activity trends across extended surfaces and different types of nanoparticles with multiple facets and defects. Such a descriptor is difficult to find, mainly because of finite-size effects. A number of theoretical^35–37^ and experimental studies^27,28^ have consistently shown those effects on the adsorption properties and ORR activities of Pt nanoparticles, but none provides a systematic way of capturing them. A simple approach for capturing finite-size effects that enables the direct comparison of nanoparticles and extended surfaces is the use of generalized coordination numbers 
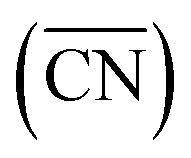
.^25,26^ Conventional coordination numbers are a count of the first nearest neighbors that captures trends on adsorption energies on extended surfaces^22–24^ but fails on nanoparticles.^35^ To be able to describe both, generalized coordination numbers weight each first-nearest neighbor atom (*j*) by its coordination number (cn(*j*)). Thus, 
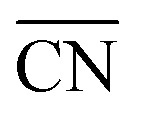
 is calculated arithmetically for a site *i* as:^25,26^
1
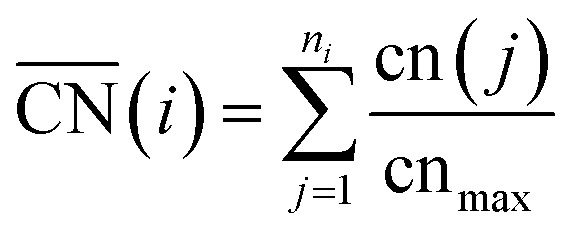



The specific value of cn_max_, which is the maximum number of first nearest neighbors in the bulk, allows 
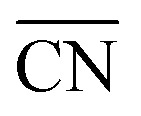
 to be defined on fcc and hcp (cn_max_ = 12) and bcc (cn_max_ = 8) crystals and guarantees that 
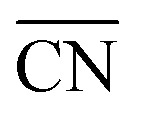
 and cn span the same ranges (0–12 for fcc and hcp crystals, and 0–8 for bcc crystals). It is clear in eqn (1) that conventional coordination numbers (cn) are a particular case of the generalized ones 
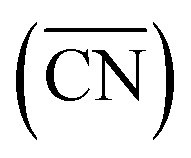
 in which all neighbors possess cn(*j*) = 12, namely the bulk coordination. Details of the assessment of 
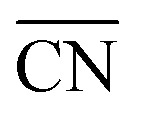
 for all sites in this study can be found in the ESI, Section S2.†

Bead-type Pt(331) (Icryst, Jülich, Germany), Pt(221), Pt(775) (both obtained from Prof. Juan Feliù, University of Alicante, Spain) and Pt(111) (Mateck, Jülich, Germany) single crystals were flame-annealed using an isobutane flame and cooled down in a 1000 ppm CO (4.7, Air Liquide, Germany) mixture with Ar (5.0, Air Liquide, Germany). The quality of the surface was checked by obtaining the characteristic voltammograms recorded in an Ar-saturated HClO_4_ electrolyte. The used electrochemical setup is described in the ESI.† All activity data were corrected for the iR-drop as described elsewhere.^38^ Further details are presented in the ESI, Section S4.†

## Results and discussion

**Fig. 1:**
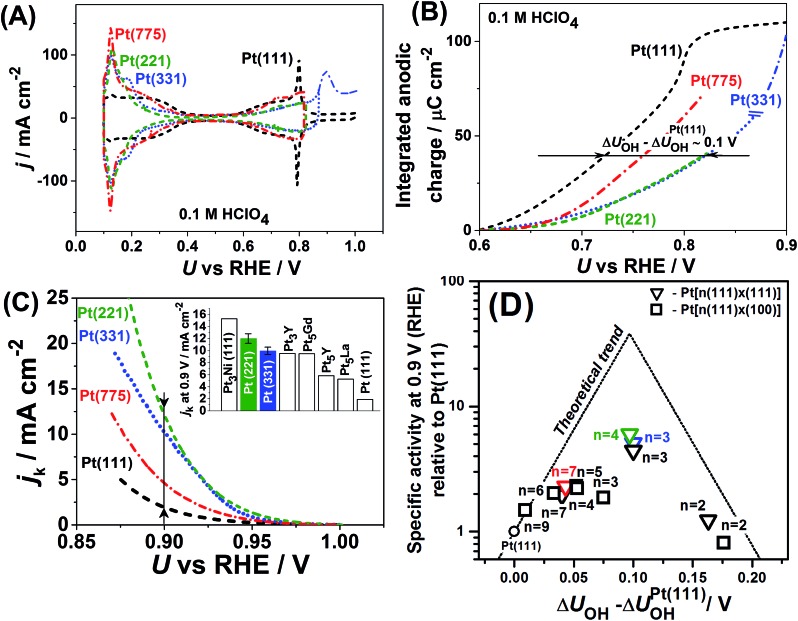
(A) Cyclic voltammograms of Pt(111), Pt(221), Pt(331) and Pt(775) in 0.1 M HClO_4_, d*U*/d*t* = 50 mV s^–1^, and (B) the integrated anodic parts of the voltammograms in which it is shown that the *OH adsorption energies for the stepped surfaces are lower with respect to Pt(111). (C) ORR-activity enhancement of Pt(221), Pt(331) and Pt(775) with respect to Pt(111) at 0.9*V*_RHE_; the inset shows a comparison of Pt(221) and Pt(331) with the most active Pt alloys reported in the literature. (D) Activity “volcano” plot for pristine Pt(111) (circle), stepped Pt[*n*(111) × (111)] (triangles) and Pt[*n*(111) × (100)] (squares) surfaces from ref. 39 and references therein are provided; data from this work (Pt(331) in blue, Pt(221) in green and Pt(775) in red) are also provided. The atomic length of the 111-terraces (*n*) is provided in each case. The data in (C) and (D) and their sources appear in Table S1.†

Fig. 1 summarizes original and literature experimental data reported by different groups on the ORR activities and *OH adsorption potentials of stepped single-crystal Pt surfaces (a similar collection of data on single-crystal Pt alloys can be found in ref. 40). Fig. 1A and B exemplify how changes in *OH adsorption potentials are assessed with respect to Pt(111) (see the ESI, Fig. S4† contains the experimental activities of single-crystal stepped Pt and ref. 39 and 41 for further details on these calculations). Fig. 1C contains the experimental activities of single-crystal stepped Pt surfaces of various (111) terrace lengths and step types ((111)-like and (100)-like), at a reference potential of 0.9*V*_RHE_. The activity trends are a function of the experimentally-assessed differences in adsorption energies of *OH with respect to Pt(111). Fig. 1C and D show that certain stepped surfaces, *e.g.* Pt(221) and Pt(331), display ORR activities that surpass those of Pt(111) and also those of most alloys, except only for Pt_3_Ni(111).

**Fig. 2:**
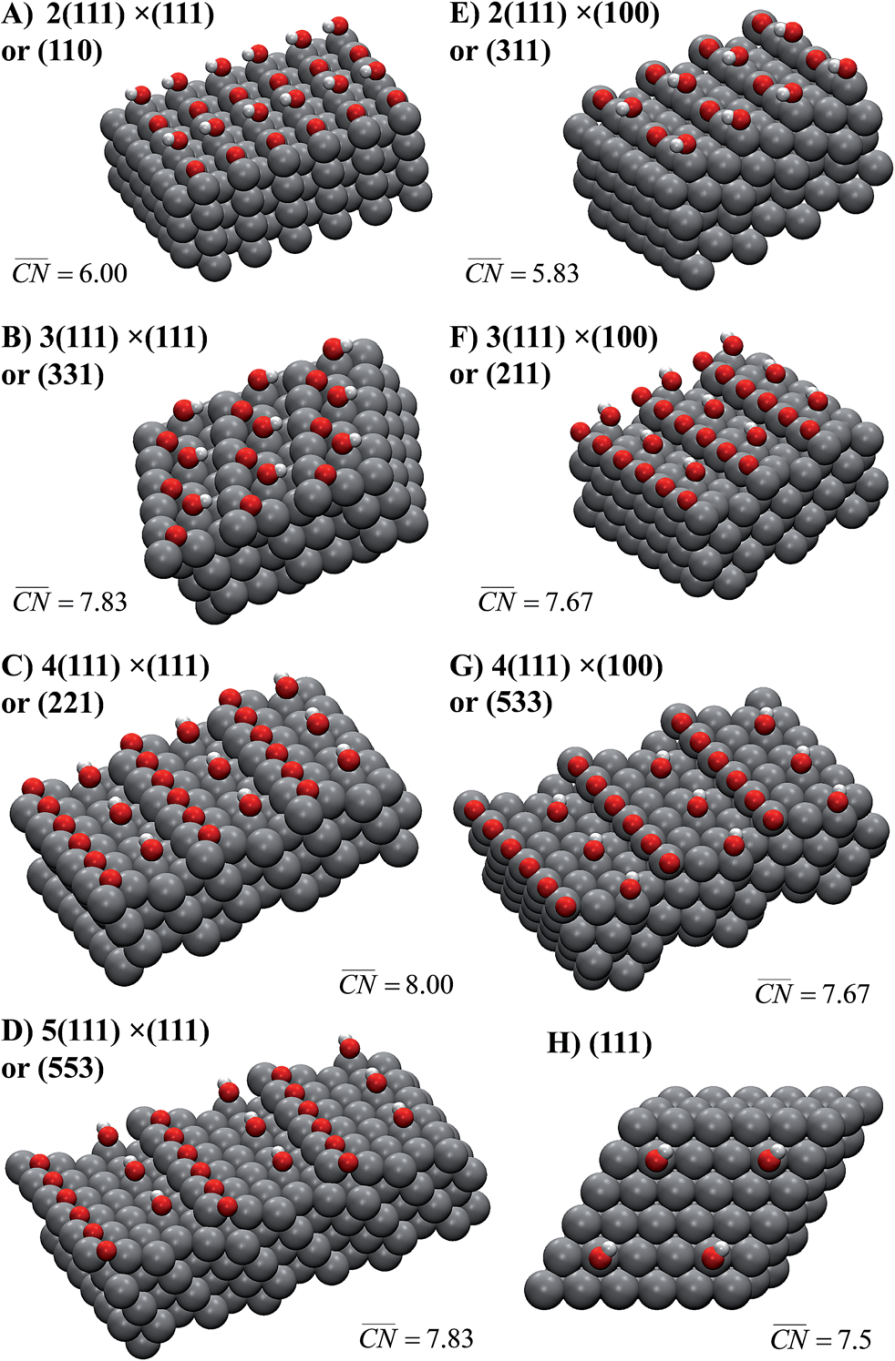
Most active sites on Pt single-crystal surfaces for the ORR. *OH sits at the active sites, which are located in all cases at the bottom of the steps (
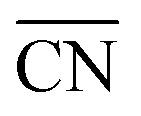
 is provided in each case, see Section S2 in the ESI† for details of its assessment). The Miller indices of the surfaces are provided together with the length of their (111) terraces and step types, namely (111)-like (left) or (100)-like (right). In all cases, the step edges are covered with *O.

Schematics of the surfaces under study appear in Fig. 2. The facets are written as Pt[*n*(*h*_1_ *k*_1_ *l*_1_) × (*h*_2_ *k*_2_ *l*_2_)]. In this notation, (*h*_1_ *k*_1_ *l*_1_) is the terrace type ((111) in all cases), *n* is the atomic length of the terraces, and (*h*_2_ *k*_2_ *l*_2_) are the step types, namely (111) or (100). For instance, Pt(221) (Fig. 2C) has 4-atom wide (111) terraces separated by (111) steps, and is denoted Pt[4(111) × (111)].

Note that at a reference potential of 0.9*V*_RHE_, although *O is likely not present at terraces (*e.g.* Fig. 2H), it does block step edges.^42,43^ Its low mobility is due to its substantial adsorption energies and relatively high diffusion barriers.^25,44^ Thus, the step edges in Fig. 2 are in all cases covered with *O to emulate the experimental conditions at 0.9*V*_RHE_. Note in passing that on surfaces with (111) steps, *O has its most stable adsorption energies on threefold (fcc) hollow sites formed by two edge Pt atoms and a terrace atom, while on those with (100) steps it prefers to adsorb on bridge sites at the edge.^25,45^ Furthermore, Fig. 2 contains the *OH adsorption sites that provide, according to our DFT calculations, the highest ORR activities on the various stepped Pt surfaces under study, while Fig. S3† contains the sites with the strongest *OH adsorption energies. In all cases, the most active adsorption sites for the ORR are located at the bottom of the steps, while the strongest adsorption sites are located at the upper edges of those steps. The corresponding potentials for *OH desorption of all sites under study are given in Table S3.† Those two types of sites coincide on the surfaces with the shortest terraces, namely Pt(110) and (311), where *n* = 2.

**Fig. 3:**
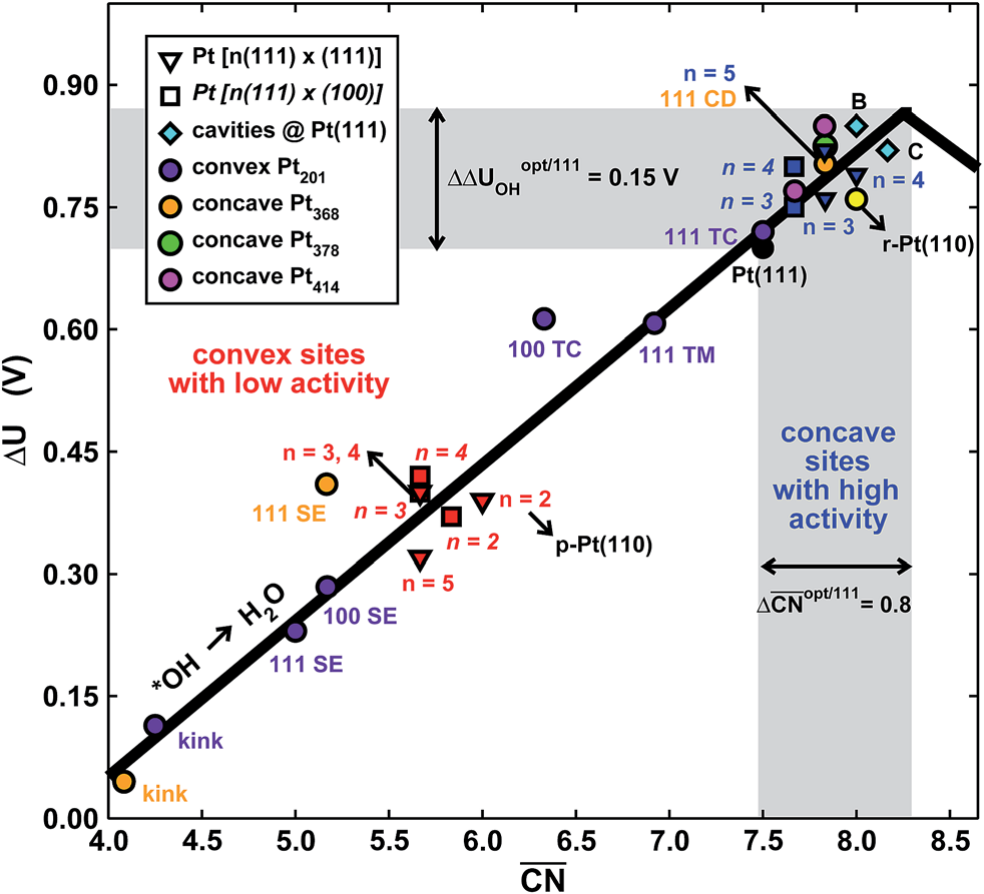
Coordination–activity plot for the ORR on Pt surfaces and nanostructures. The plot correlates 
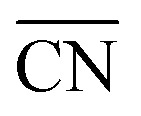
 with the potentials of the ORR limiting steps. The ORR overpotential is the difference between 1.23 V and those potentials. Convex sites 
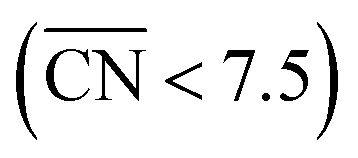
 located *e.g.* at step edges (red) have larger overpotentials than Pt(111), while concave sites 
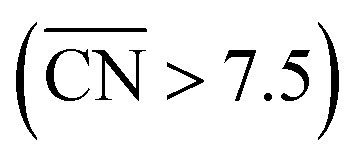
 at *e.g.* the bottom of steps (blue) have lower overpotentials. Data are included for Pt[*n*(111) × (111)] (), Pt[*n*(111) × (100)] (■, italics), missing-row-reconstructed Pt(110) (yellow, denoted r-Pt(110)), and cavities on Pt(111) (♦) from ref. 30, and one convex and three concave nanoparticles (Pt_201_, Pt_368_, Pt_378_, Pt_414_). TC: terrace center; TM: terrace middle; SE: step edge; CD: concave defect. See Fig. 4, S9, S10 and Table S3† for further details. 
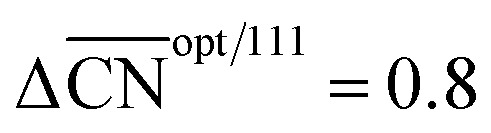
 and ΔΔ*U* ^opt/111^ = 0.15 V delimit the energetic-coordination region (in gray) of improvement with respect to Pt(111).

To assess the individual activity of the various sites in Fig. 2 and S3,† we constructed the coordination–activity plot in Fig. 3 (see also Fig. S10†). In this graph we include the activities of both the most active sites on the bottoms of the steps (blue points in the gray area) and the inactive ones at their edges (red points in the lower left). Note that the generalized coordination number of any surface Pt atom at a pristine (111) surface is 
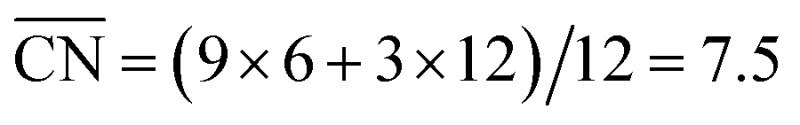
. Since all of the points are located on the left side of the volcano plot, simple and general conclusions can be obtained: sites with 
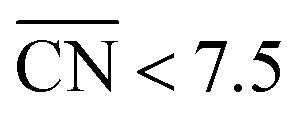
 bind *OH stronger than Pt(111) and have larger overpotentials, while those with 
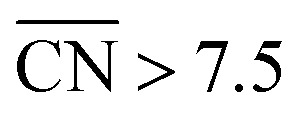
 bind *OH weaker than Pt(111) (by no more than 0.15 V, as predicted using conventional volcano plots^11^) and have smaller overpotentials. This means that the introduction of undercoordinated sites on Pt(111) does not enhance its catalytic activity. The enhancement comes from the highly coordinated sites formed in the vicinity of those undercoordinated sites (see Fig. 2 and S3†). Such highly coordinated sites do not form at the surface of convex nanoparticles, which explains in simple terms their lower activities compared to Pt(111) electrodes. That is why creating atomic-scale cavities in Pt(111) enhances the ORR electrocatalysis, without any need for alloying (see ref. 30 and data points B and C in Fig. 3, which correspond to overcoordinated Pt(111) sites at the bottom of such cavities). As the differences in Fig. 3 between step-edge and step-bottom sites are as large as ∼0.4 V in ORR overpotentials and ∼2 units in coordination, clear distinctions exist between inactive and active sites within this model.

Note that Fig. 1D shows that Pt[2(111) × (111)] (*i.e.* Pt(110)) is 20% more active than Pt(111), which is in line with previous work.^46^ The coordination–activity plot in Fig. 3 predicts that pristine Pt(110) (denoted p-Pt(110)) is not active for the ORR. However, it is well known that Pt(110) reconstructs under electrochemical conditions^47–50^ in a missing-row fashion (see Fig. S8†). The missing-row structure, for which we have included data in Fig. 3 (r-Pt(110), yellow), possesses wider terraces where sites with 
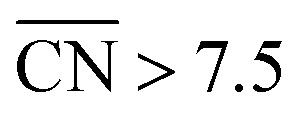
 are present (see Fig. S2†) and are responsible for the activity enhancement with respect to Pt(111). This coincides with recent measurements by Attard and Brew, who found the following ORR activity ordering: p-Pt(110) < Pt(111) < r-Pt(110).^50^

A noteworthy feature of Fig. 1C is the unusual dependence of the kinetic current on the potential for Pt(331). While Pt(111), Pt(775) and Pt(221) exhibit nearly exponential growth and the difference in activities increases with the overpotential, Pt(331) loses its high activity. This can be understood as follows: on this surface, *O makes an important contribution to the nearest-neighbor counting of the most active sites due to the short terrace length and the threefold adsorption configuration of *O (Fig. 2B). Under ORR conditions, adsorbate coverage depends on the electrode potential in a way such that at high potentials, high *O coverages are observed. As the potential is lowered, the *O coverage is lowered, which decreases the generalized coordination number of the active sites from 7.83 to 7.5 when all *O is reduced. Conversely, surfaces with larger terraces such as Pt(221) and Pt(775) do not depend on the presence of *O at steps to have highly coordinated sites at their step bottoms and maintain their high activity, so that a quasi-exponential current growth is observed.

In summary, the coordination–activity plot in Fig. 3 (see also Fig. S10†) is a useful tool to determine the relationship between the activity and geometry of different types of active sites. In particular for the ORR on Pt, the high activity region corresponds to highly coordinated sites located at concavities, which explains why stepped Pt surfaces are more active than Pt(111). Note, however, that the overall catalytic activity of a given surface will not only depend on the presence of those sites, but also on their relative abundance.

Aiming at connecting model-surface and nanoparticle design principles, we will now extend the approach based on 
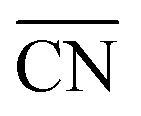
 to nanoparticles of various shapes.

**Fig. 4:**
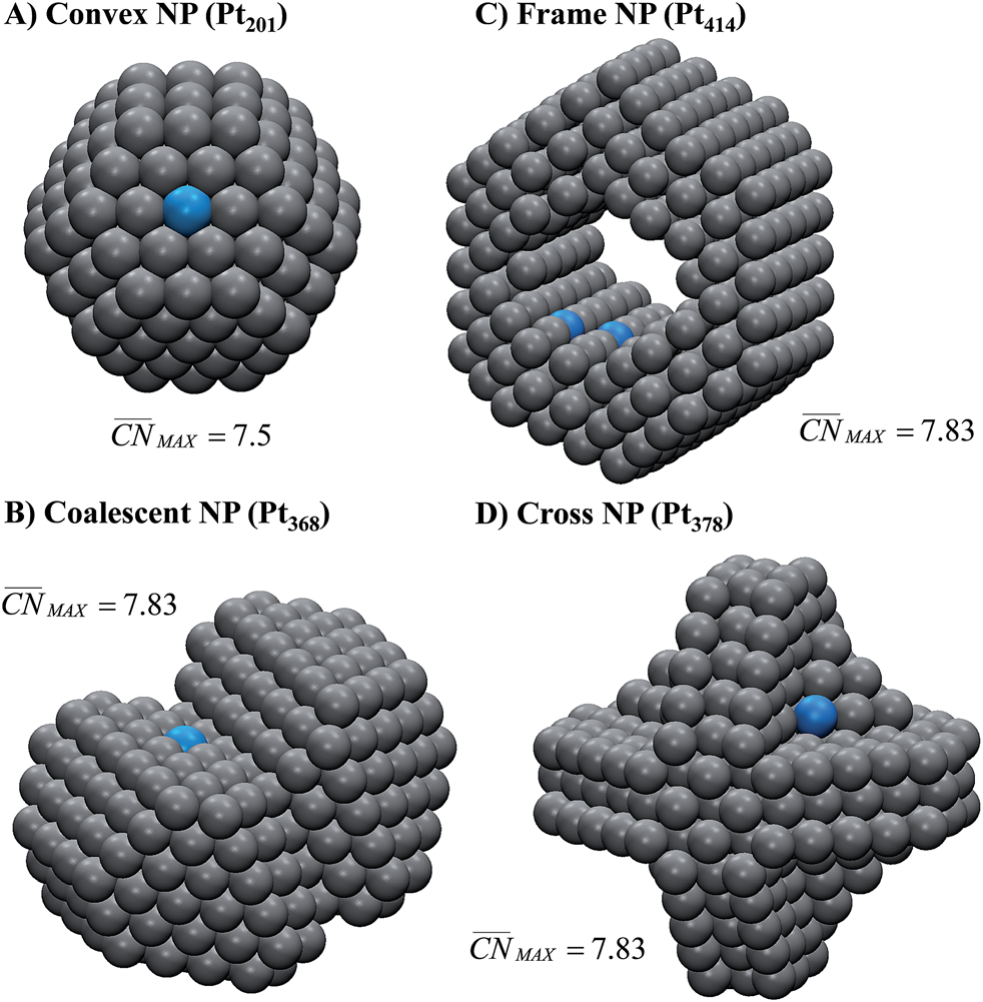
Most active sites on various Pt nanostructures. (A) “Classical” convex nanoparticle; (B) coalescent convex nanoparticle, at the contact of which a concavity is formed; (C) frame nanoparticle; (D) cross nanoparticle. The sites with the largest 
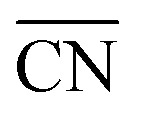
 and nearly optimal *OH adsorption energies appear in blue and their activities appear in Fig. 3. More examples (superstructures/high loading of nanoparticles or idealized porous films) are given in the ESI, Section S5.†

Let us start the analysis with Pt_201_, a typical convex nanoparticle with only (111) and (100) terraces present at the surface, as shown in Fig. 4A. 
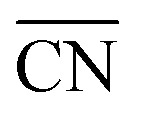
 is maximal (7.5) for atop sites at the center of the (111) terraces, while the sites closer to the edges or at the (100) terraces have lower coordination (see Fig. S9†).^25,26^ Pt_201_ in Fig. 4A is a truncated octahedron, but this conclusion based on 
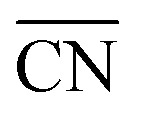
 holds for any other convex particle shape, for instance, a regular octahedron, cuboctahedron, *etc.* For all those convex shapes, the upper limit for 
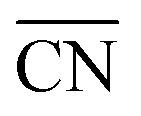
 is 7.5. In small particles, this value is only reached by a small fraction of surface atoms, located at the center of (111) terraces as the blue atom in Fig. 4A. Therefore, convex individual Pt nanoparticles cannot be substantially more active than Pt(111), as shown in Fig. 3, where the ORR overpotentials for all sites on Pt_201_ (and two convex defects on Pt_368_) are equal to or larger than those of Pt(111). Indeed, numerous experimental data confirm this claim: with the increase of the particle size, the specific ORR activity (*i.e.* the activity normalized per real surface area) of convex nanoparticles only approaches the activity of bulk Pt electrodes.^27,28^ This trend is justified by the increasing fraction of (111) facets in the particles.^36,37^

Our next example considers two convex nanoparticles in contact with each other (see Fig. 4B, the orange data in Fig. 3 and S6†). This situation may be found in supported electrocatalysts with relatively large loadings of Pt nanoparticles when these are in contact with each other but do not aggregate (note that when the particles are not in contact but their double layers overlap the ORR is also enhanced).^51^ Furthermore, this type of contact/coalescence may also be found in the ordered arrays of nanoparticles recently synthesized and found to be highly active for the ORR.^20^ As shown in Fig. 4B, the partial coalescence of two or more convex particles results in a larger, concave particle. At the concavity, sites with 
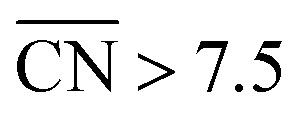
 are formed that possess smaller overpotentials compared to Pt(111), while convex defects such as edges and kinks on the same particle are considerably less active, see Fig. 3 and Table S3.† Thus, our model suggests that one can enhance the activity of Pt nanoparticles in two ways: (i) increasing the nanoparticle loading while avoiding significant aggregation,^51^ and (ii) creating ordered nanoparticle arrays, which can extend on 1D (chains) or 2D (grids).^20^

Fig. 4C shows an example of the frame nanoparticles that are promising to increase the mass activity of Pt-based ORR electrocatalysts due to their high surface-to-volume ratio. This type of concave morphology also possesses two types of sites for which 
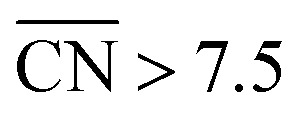
 and hence the predicted overpotentials are smaller than those of Pt(111) (see Table S3†). Again, recent experimental measurements have confirmed the exceptionally high ORR specific activity of these nanoparticles.^17–19^ Furthermore, Fig. 3 and 4C show that cross-shaped nanoparticles, the anisotropic growth of which has been studied by Strasser and coworkers,^52^ also possess active concave sites for the ORR.

Finally, a way of improving the ORR activity of Pt electrocatalysts is the design of mesostructured films. The preparation of pores with specific arrangements (see schematics in Fig. S7†) that create sites with 
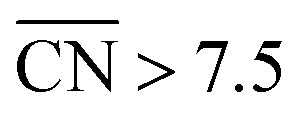
 would be of particular interest to obtain more active Pt-based materials with increased activity and stability. Remarkably, mesostructured films do demonstrate surprisingly high ORR activities^21^ that can also be understood in terms of 
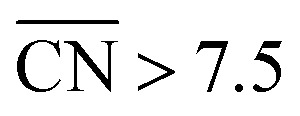
, which is also the case of the highly active Pt-based nanoparticles with microstrain recently reported by Chattot *et al.* ^29^

## Conclusions

Careful model-surface observations obtained from single-crystal experiments do not necessarily lead to the design of enhanced Pt nanoparticle catalysts for the ORR. This is because stepped single-crystal surfaces contain both convex (step edges) and concave sites (step bottoms), but convex nanoparticles contain only the former. While convex defects are not active for the ORR, concave defects are very active and can outperform sites on pristine Pt(111) if they do not have steric hindrance. Concave defects are not present in convex nanoparticles, explaining why the activity of this type of particle increases alongside the number of (111) terrace sites. Concavities are, however, present in a variety of active nanostructures.

**Fig. 5:**
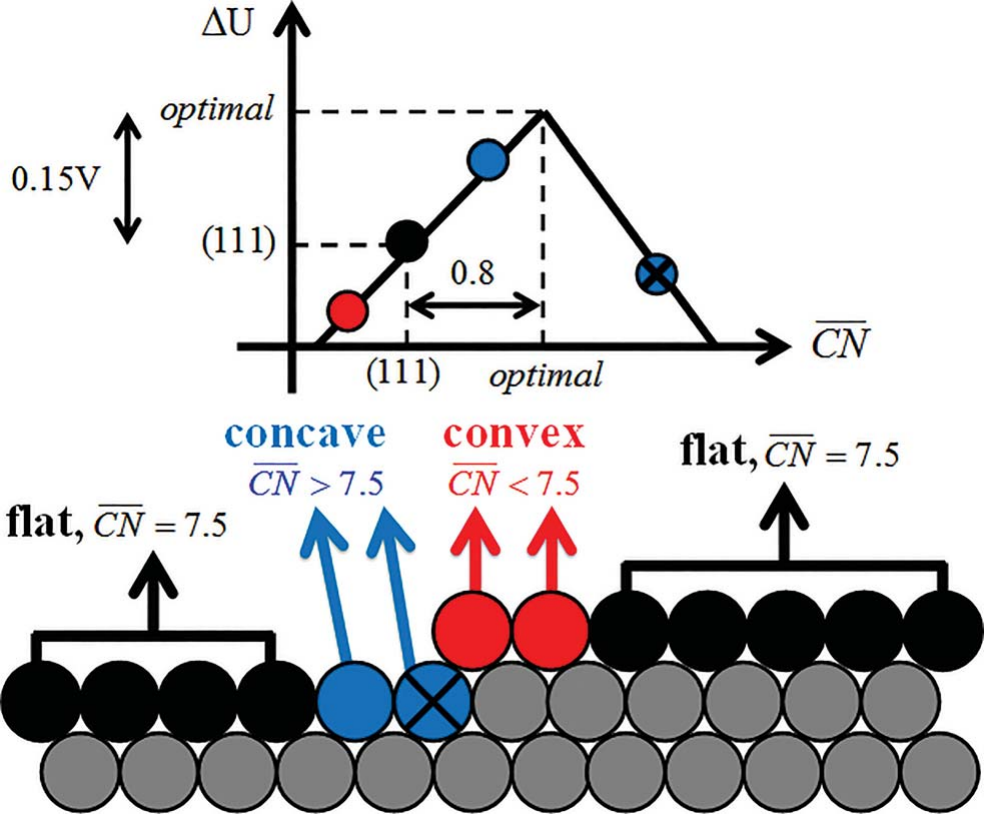
Summary of the predictions of the coordination–activity plot and their relationship to the geometry of Pt sites. Four types of sites exist: flat (111) sites (black); convex sites (red) that are less active than flat sites; concave sites (blue) that are more active than flat sites; and concave sites with steric hindrance (blue cross). The optimal catalyst is simultaneously more coordinated than Pt(111) 
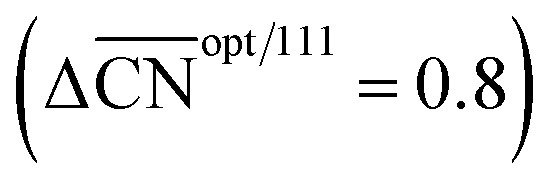
 and binds *OH more weakly (ΔΔ*U* ^opt/111^ = 0.15 V).

Our results suggest that, in general, active ORR sites possess two distinctive and equivalent features: they bind *OH up to ∼0.1–0.15 V more weakly than Pt(111) and possess 
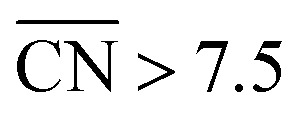
. These conclusions are summarized in Fig. 5, where there is an explicit connection between the geometry, adsorption energy and ORR activity of Pt sites, which explains the high activity of a wide variety of Pt single-crystal electrodes and nanostructures.

Coordination–activity plots can also be used to design ORR catalysts made of metals other than Pt such as Au,^30^ and for other electrocatalytic reactions such as the hydrogen evolution reaction.^53^

## Note added after first publication

This article replaces the version published on 19th December 2016, in which an incorrect version of Fig. 5 was presented through editorial error.

## References

[cit1] Ertl G. (2008). Angew. Chem., Int. Ed..

[cit2] Jaramillo T. F., Jørgensen K. P., Bonde J., Nielsen J. H., Horch S., Chorkendorff I. (2007). Science.

[cit3] Koper M. T. M. (2011). Nanoscale.

[cit4] Nørskov J. K., Bligaard T., Rossmeisl J., Christensen C. H. (2009). Nat. Chem..

[cit5] O'Mullane A. P. (2014). Nanoscale.

[cit6] Chorkendorff I. and Niemantsverdriet H., Concepts of Modern Catalysis and Kinetics, Wiley, New York, 2006.

[cit7] Thomas J. M. and Thomas W. J., Principles and Practice of Heterogeneous Catalysis, Wiley, New York, 1996.

[cit8] Gasteiger H. A., Marković N. M. (2009). Science.

[cit9] Rabis A., Rodriguez P., Schmidt T. J. (2012). ACS Catal..

[cit10] Vesborg P. C. K., Jaramillo T. F. (2012). RSC Adv..

[cit11] Stephens I. E. L., Bondarenko A. S., Gronbjerg U., Rossmeisl J., Chorkendorff I. (2012). Energy Environ. Sci..

[cit12] Greeley J., Stephens I. E. L., Bondarenko A. S., Johansson T. P., Hansen H. A., Jaramillo T. F., Rossmeisl J., Chorkendorff I., Nørskov J. K. (2009). Nat. Chem..

[cit13] Climent V., Feliu J. M. (2011). J. Solid State Electrochem..

[cit14] Hitotsuyanagi A., Nakamura M., Hoshi N. (2012). Electrochim. Acta.

[cit15] Kuzume A., Herrero E., Feliu J. M. (2007). J. Electroanal. Chem..

[cit16] Stamenkovic V. R., Fowler B., Mun B. S., Wang G., Ross P. N., Lucas C. A., Marković N. M. (2007). Science.

[cit17] Chen C., Kang Y., Huo Z., Zhu Z., Huang W., Xin H. L., Snyder J. D., Li D., Herron J. A., Mavrikakis M., Chi M., More K. L., Li Y., Markovic N. M., Somorjai G. A., Yang P., Stamenkovic V. R. (2014). Science.

[cit18] Dubau L., Lopez-Haro M., Durst J., Guetaz L., Bayle-Guillemaud P., Chatenet M., Maillard F. (2014). J. Mater. Chem. A.

[cit19] Becknell N., Kang Y., Chen C., Resasco J., Kornienko N., Guo J., Markovic N. M., Somorjai G. A., Stamenkovic V. R., Yang P. (2015). J. Am. Chem. Soc..

[cit20] Kang Y., Ye X., Chen J., Cai Y., Diaz R. E., Adzic R. R., Stach E. A., Murray C. B. (2013). J. Am. Chem. Soc..

[cit21] Kibsgaard J., Gorlin Y., Chen Z., Jaramillo T. F. (2012). J. Am. Chem. Soc..

[cit22] Calle-Vallejo F., Loffreda D., Koper M. T. M., Sautet P. (2015). Nat. Chem..

[cit23] Jiang T., Mowbray D. J., Dobrin S., Falsig H., Hvolbæk B., Bligaard T., Nørskov J. K. (2009). J. Phys. Chem. C.

[cit24] Li H., Li Y., Koper M. T. M., Calle-Vallejo F. (2014). J. Am. Chem. Soc..

[cit25] Calle-Vallejo F., Martínez J. I., García-Lastra J. M., Sautet P., Loffreda D. (2014). Angew. Chem., Int. Ed..

[cit26] Calle-Vallejo F., Sautet P., Loffreda D. (2014). J. Phys. Chem. Lett..

[cit27] Perez-Alonso F. J., McCarthy D. N., Nierhoff A., Hernandez-Fernandez P., Strebel C., Stephens I. E. L., Nielsen J. H., Chorkendorff I. (2012). Angew. Chem., Int. Ed..

[cit28] Shao M., Peles A., Shoemaker K. (2011). Nano Lett..

[cit29] Chattot R., Asset T., Bordet P., Drnec J., Dubau L., Maillard F. (2017). ACS Catal..

[cit30] Calle-Vallejo F., Tymoczko J., Colic V., Vu Q. H., Pohl M. D., Morgenstern K., Loffreda D., Sautet P., Schuhmann W., Bandarenka A. S. (2015). Science.

[cit31] Kresse G., Furthmüller J. (1996). Phys. Rev. B: Condens. Matter.

[cit32] Perdew J. P., Burke K., Ernzerhof M. (1996). Phys. Rev. Lett..

[cit33] Kresse G., Joubert D. (1999). Phys. Rev. B: Condens. Matter.

[cit34] Nørskov J. K., Rossmeisl J., Logadottir A., Lindqvist L., Kitchin J. R., Bligaard T., Jónsson H. (2004). J. Phys. Chem. B.

[cit35] Kleis J., Greeley J., Romero N. A., Morozov V. A., Falsig H., Larsen A. H., Lu J., Mortensen J. J., Dułak M., Thygesen K. S., Nørskov J. K., Jacobsen K. W. (2011). Catal. Lett..

[cit36] Tripković V., Cerri I., Bligaard T., Rossmeisl J. (2014). Catal. Lett..

[cit37] Tritsaris G. A., Greeley J., Rossmeisl J., Nørskov J. K. (2011). Catal. Lett..

[cit38] Čolić V., Tymoczko J., Maljusch A., Ganassin A., Schuhmann W., Bandarenka A. S. (2015). ChemElectroChem.

[cit39] Bandarenka A. S., Hansen H. A., Rossmeisl J., Stephens I. E. L. (2014). Phys. Chem. Chem. Phys..

[cit40] Čolić V., Bandarenka A. S. (2016). ACS Catal..

[cit41] Rossmeisl J., Karlberg G. S., Jaramillo T., Nørskov J. K. (2009). Faraday Discuss..

[cit42] Casalongue H. S., Kaya S., Viswanathan V., Miller D. J., Friebel D., Hansen H. A., Nørskov J. K., Nilsson A., Ogasawara H. (2013). Nat. Commun..

[cit43] Pohl M. D., Colic V., Scieszka D., Bandarenka A. S. (2016). Phys. Chem. Chem. Phys..

[cit44] Peng G., Mavrikakis M. (2015). Nano Lett..

[cit45] Kolb M. J., Calle-Vallejo F., Juurlink L. B. F., Koper M. T. M. (2014). J. Chem. Phys..

[cit46] Markovic N. M., Gasteiger H. A., Ross P. N. (1995). J. Phys. Chem..

[cit47] Hoshi N., Nakamura M., Sakata O., Nakahara A., Naito K., Ogata H. (2011). Langmuir.

[cit48] Lucas C. A., Marković N. M., Ross P. N. (1996). Phys. Rev. Lett..

[cit49] Marković N. M., Grgur B. N., Lucas C. A., Ross P. N. (1997). Surf. Sci..

[cit50] Attard G. A., Brew A. (2015). J. Electroanal. Chem..

[cit51] Nesselberger M., Roefzaad M., Fayçal Hamou R., Ulrich Biedermann P., Schweinberger F. F., Kunz S., Schloegl K., Wiberg G. K. H., Ashton S., Heiz U., Mayrhofer K. J. J., Arenz M. (2013). Nat. Mater..

[cit52] Gan L., Cui C., Heggen M., Dionigi F., Rudi S., Strasser P. (2014). Science.

[cit53] Tymoczko J., Calle-Vallejo F., Schuhmann W., Bandarenka A. S. (2016). Nat. Commun..

