# Supplementary material for: Why conclusions from platinum model surfaces do not necessarily lead to enhanced nanoparticle catalysts for the oxygen reduction reaction
Source: Chem Sci. 2016 Dec 6;8(3):2283–9. doi: 10.1039/c6sc04788b (PMC5363395; doi:10.1039/c6sc04788b)
Supplement: Supplementary file 1 [file SC-008-C6SC04788B-s001.pdf]

Electronic Supplementary Material (ESI) for Chemical Science.
This journal is © The Royal Society of Chemistry 2016

Electronic Supplementary Information for:

Why conclusions from platinum model surfaces do not necessarily lead to enhanced nanoparticle catalysts for the oxygen reduction reaction

Federico Calle-Vallejo,[1,] Marcus D. Pohl,[2] David Reinisch,[2] David Loffreda,[3] Philippe Sautet,[3,4] Aliaksandr S. Bandarenka[2,5,*]*

[1] Leiden Institute of Chemistry, Leiden University, PO Box 9502, 2300 RA Leiden, The Netherlands.

[2] Physik-Department ECS,Technische Universität München, James-Franck-Str. 1, D-85748 Garching, Germany.

[3] Univ Lyon, Ens de Lyon, CNRS UMR 5182, Université Claude Bernard Lyon 1, Laboratoire de Chimie, F-69342, Lyon, France.

[4] Department of Chemical and Biomolecular engineering, University of California, Los Angeles, Los Angeles, CA 90095, United States.

[5] Nanosystems Initiative Munich (NIM), Schellingstraße 4, 80799 Munich, Germany.

* Corresponding Authors: f.calle.vallejo@lic.leidenuniv.nl, bandarenka@ph.tum.de

Table of contents

S1. Experimental activity data S2

S2. Additional theoretical details S2

S3. Experimental assessment of *OH adsorption energies S8

S4. Electrochemical measurements S8

S5. Generalized coordination numbers for some other electrocatalytic systems S9

S6. Concave region of the coordination-activity plot S12

References S12

S1. Experimental activity data

In Table S1 we provide the data used in Figure 1 in the main text.

Table S1. Experimental ORR activities for stepped Pt single-crystal electrodes with (111) terraces of various lengths separated by (111) or (100) steps. The most active surfaces are highlighted in grey.

Surface	Experimental ORR activity improvement over Pt(111) at 0.9 V_{RHE}	Experimental ORR activity expressed as the "half-wave" potential, $E_{1/2}$ (V)	References
**Pt(111)**	1	~0.864	1,2,3
**Pt[2(111)×(111)]**	~1.2	~0.876	3
**Pt[3(111)×(111)]**	~4.45	~0.909	3
**Pt[4(111)×(111)]**	-	~0.907	3
**Pt[2(111)×(100)]**	~0.82		2
**Pt[3(111)×(100)]**	~1.9		2
**Pt[4(111)×(100)]**	~2.1		2
**Pt[3(111)×(111)]**	~5.4	~0.912	This work
**Pt[4(111)×(111)]**	~6.2	~0.917	This work
**Pt[7(111)x(111)]**	~2.4	~0.893	This work

S2. Additional theoretical details

In the model by Norskov et al [4], the energetics of protons and electrons in electrochemical environments are approximated by those of hydrogen gas, according to the following equilibrium:

$$\tfrac{1}{2}H_2 \leftrightarrow H^+ + e^-$$ * MERGEFORMAT (S1)

In addition, we used the reaction mechanism below to model ORR:

$$* + O_2 + H^+ + e^- \rightarrow *OOH$$ * MERGEFORMAT (S2)

$$*OOH + H^+ + e^- \rightarrow *O + H_2O$$ * MERGEFORMAT (S3)

$$*O + H^+ + e^- \rightarrow *OH$$ * MERGEFORMAT (S4)

$$*OH + H^+ + e^- \rightarrow * + H_2O$$ * MERGEFORMAT (S5)

Where * denotes a free adsorption site. The two potential-determining steps in this mechanism are typically the first one, namely O_2 adsorption and protonation to produce *OOH, and the fourth one, namely *OH protonation to produce H_2O [4, 5]. Only the latter step is potential-determining for the Pt catalysts under study, as all of them have $\overline{CN} < 8.3$ (see Figures 2 and 3 in the main text). The free energy of reaction of this step is calculated as follows:

$$\Delta G_4 = -\Delta G_{OH} \quad \text{* MERGEFORMAT (S6)}$$

The adsorption energy of *OH is:

$$\Delta G_{OH} = G_{*OH} + \tfrac{1}{2} G_{H_2} - G_{*} - G_{H_2O} \quad \text{* MERGEFORMAT (S7)}$$

We approximate the free energies of the adsorbates as:

$$G \approx E_{DFT} + ZPE - TS + E_{solvation} \quad \text{* MERGEFORMAT (S8)}$$

E_{DFT} is the DFT-calculated total energy. *ZPE* is the zero-point energy estimated through vibrational-frequency analysis within the harmonic-oscillator approximation. $E_{solvation}$ is the solvation energy granted by the liquid, which is -0.575 for *OH, in line with previously reported values [5,6,12]. Entropy corrections for adsorbed species were taken to be zero, so that our results can be directly compared to other studies in the literature [5,7]. Reaction energies (ΔG in eV) and electrochemical reduction potentials (ΔU in V) are related as: $\Delta U = -\Delta G / n_e$, where n_e is the number of transferred electrons. G_{H_2O} and G_{H_2} were also estimated with equation * MERGEFORMAT (S8). In order to represent liquid-phase water, *TS* is that of the gas phase and $E_{solvation}$ is the difference between the formation energies of gas-phase and liquid-phase water, namely -0.087 eV at 298 K [4,8]. For H_2, $E_{solvation}$ is zero, and TS is taken from thermodynamic tables. All corrections used in this study appear in Table S2.

Table S2. Zero-point-energy (*ZPE*), entropy (*TS*) and solvation ($E_{solvation}$) corrections in eV at 298.15 K for various species involved in this study.

Species	*ZPE*	*TS*	$E_{solvation}$
H_2(g)	0.270	0.404	-
H_2O(l)	0.574	0.583	-0.087
*OH	0.332	-	-0.575

Regarding the assessment of generalized coordination numbers, first of all, we note that as *OH is the archetypal ORR intermediate (justifying the choice of activity descriptor in Figure 1 in the main text), it is important to elucidate the qualitative and quantitative aspects of its adsorption on Pt surfaces. At the reference electrode potential of 0.9 V_{RHE}, ideally flat Pt(111) surfaces adsorb ~1/3 monolayer (ML) of *OH in cyclic voltammetry [9]. Besides, recent findings (see [10,11,12,13]) strongly suggest that *OH is an atop adsorbate on Pt(111) embedded in a half-dissociated water layer. Those two conditions can be modelled through a surface lattice of the type $\sqrt{3}\times\sqrt{3}\,R30°$ (see Figure S1), in which every *OH adsorbate is surrounded by water only and its coverage is 1/3 ML. Importantly, we do not include *H_2O species in the calculation of $\overline{CN}$ in view of their weak adsorption energies on Pt extended surfaces and nanoparticles [14].

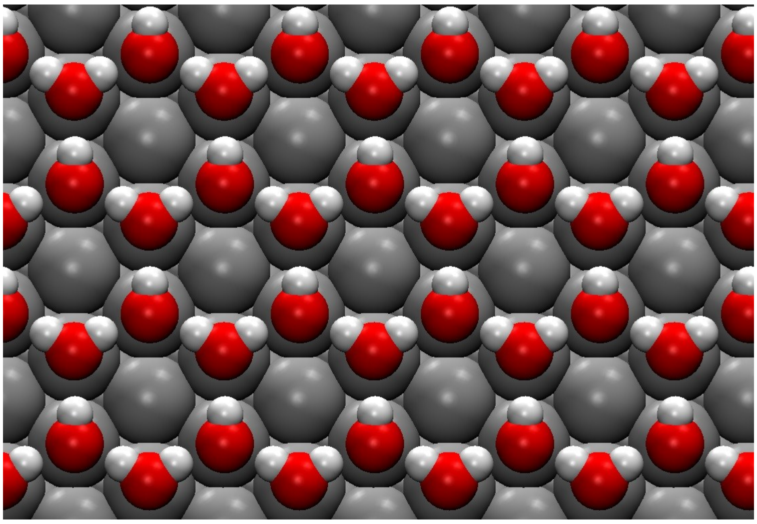

Figure S1. Pt(111) surface with the typical hexagonal H_2O/*OH bilayer. The coverage of *OH is 1/3 ML.

At 0.9 V_{RHE}, defective Pt surfaces such as stepped single crystals adsorb not only *OH but also *O. The latter adsorbate is supposed to be located at the step edges, while the (111) terraces will tend to form a half-dissociated water layer, the extension of which depends on the terrace width. Figure 2 in the main text gives an overview of the most probable *O adsorption sites on various stepped Pt surfaces. At these electrode potentials, *O blocks step edges. Its low mobility is due to its substantial adsorption energies and relatively high diffusion barriers [15]. Besides, *O is not part of the (half-dissociated) water layer due to its poor solvation and adsorption configuration (threefold hollow sites on terraces, and bridge sites at step edges, while adsorbates at water layers are atop) [12,13]. In view of these features, *O is included in the calculation of

$\overline{CN}$ for the neighboring sites. However, as can be seen in Table S3, accounting for *O is necessary only to explain the high activity of Pt(331), as step-bottom sites at surfaces with longer terraces are not affected by its presence. Therefore, including or omitting *O in the assessment of $\overline{CN}$ does not change the overall conclusions of this study. *An example of the count with *O is given below in Figure S2, where $\overline{CN}$ of an *OH adsorption site on missing-row Pt(110) is detailed.

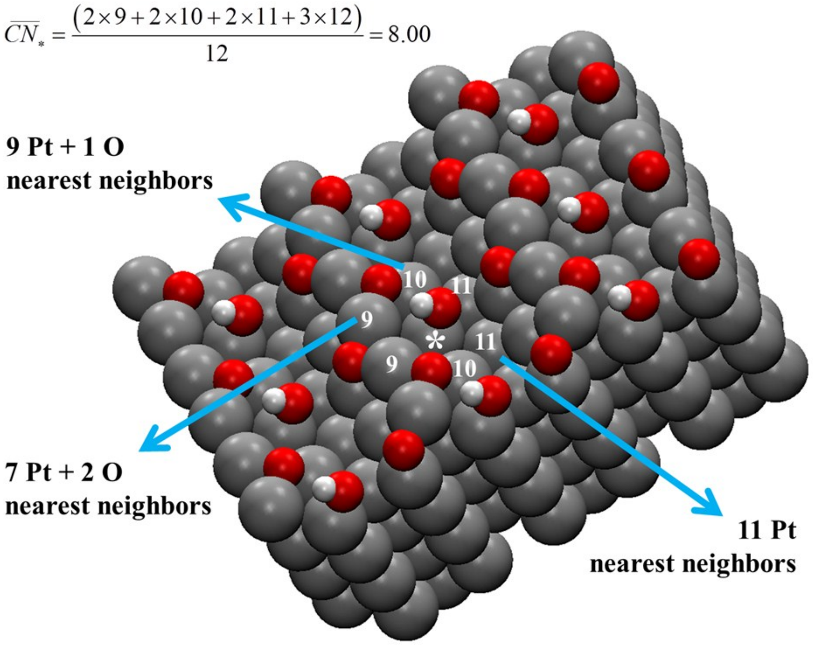

Figure S2. Schematics of the missing-row reconstruction of Pt(110) with coadsorbed *O and *OH. The generalized coordination number of the site on which *OH adsorbs (marked with *) is provided and the way of accounting for the nearest neighbors of the first-nearest neighbors is specified. There are 3 nearest neighbors in the subsurface with 12 Pt nearest neighbors.

Figure S3 contains the most stable coadsorption configurations of *O and *OH on the stepped surfaces under study. $\overline{CN}$ of the Pt atoms over which *OH is adsorbed are also provided. In all cases, *OH is located at step edges and *O is adsorbed at the same sites as in Figure 2 in the main text, though its coverage is lower due to the site competition with *OH. The common feature of the sites in panels A-G in Figure S2 is that $\overline{CN} < 7.5$, due to the low coordination of atoms at step edges and their vicinities.

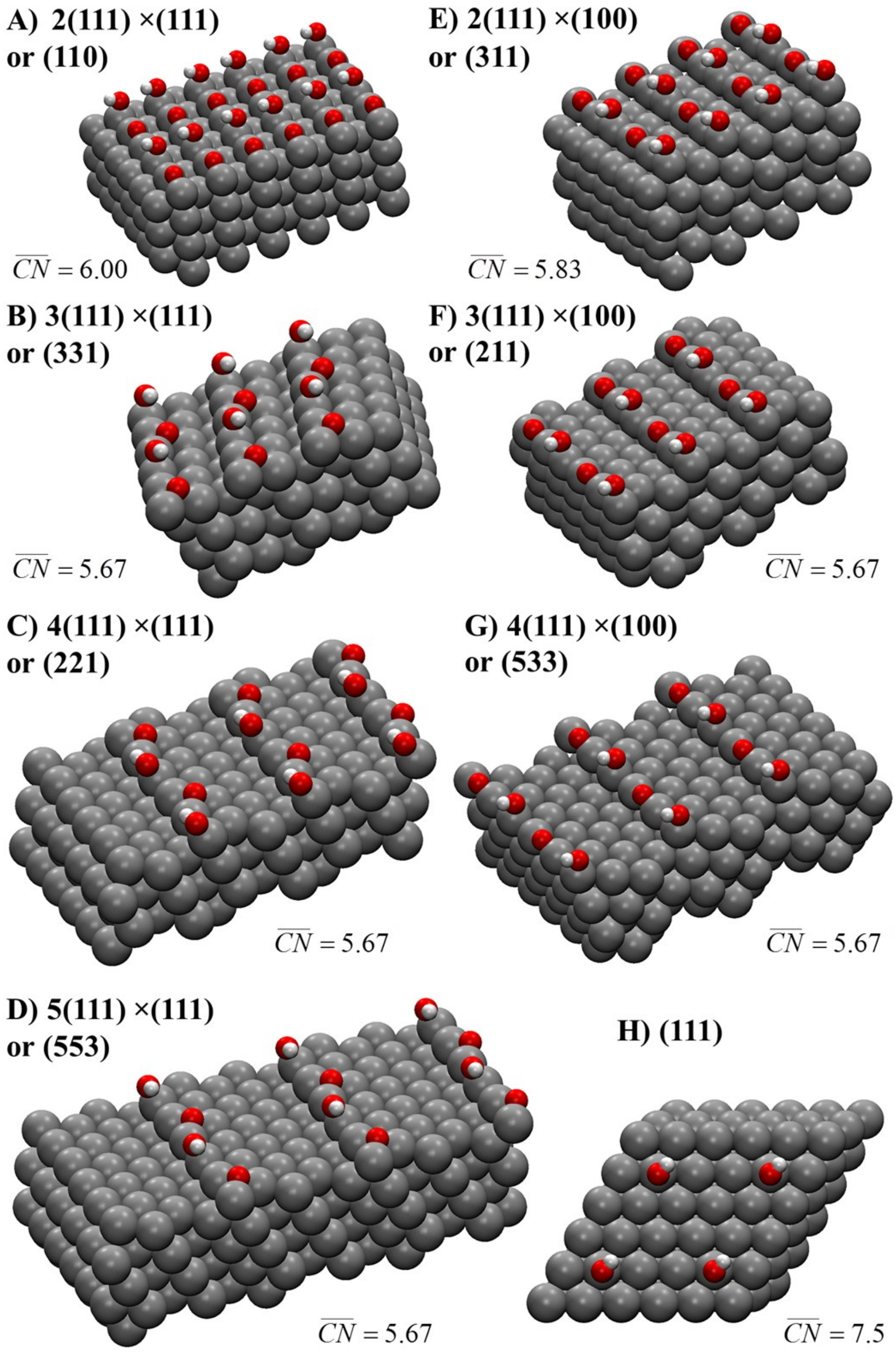

Figure S3. Strongest atop adsorption sites for *OH on various Pt single-crystal surfaces. The generalized coordination numbers of the sites are provided in each case. All surfaces contain only (111) terraces of various lengths, separated by (111) (left) or (100) (right) steps. The Miller indices, terrace lengths and step types are provided in each case.

The data used to build Figure 3 in the main text are provided in Table S3. It contains the site descriptions of Figures 2, 4, S3 and S8 and the potentials for *OH transformation into H_2O, which is the potential-limiting step for the ORR on the Pt catalysts under study.

Table S3. Surface sites under study and their calculated potential-limiting steps for the ORR. SB: step bottom. SE: step edge. p-(110): pristine (110) facet. r-(110): missing-row reconstructed (110) facet. TC and TM: terrace center and middle. CD: concave defect. $\overline{CN}*$: *O is omitted in the nearest-neighbor counting.

Surface	step type	n	$\overline{CN}$	$\overline{CN}*$	ΔU / V $(*OH \rightarrow H_2O)$
(111)	-	-	7.50	7.50	0.70
(311)	square	2	5.83	5.50	0.37
(211) SB	square	3	7.67	7.33	0.75
(211) SE	square	3	5.67	5.50	0.40
(533) SB	square	4	7.67	7.67	0.80
(533) SE	square	4	5.67	5.50	0.42
p-(110)	triangular	2	6.00	5.83	0.39
r-(110)* SB	triangular	3	8.00	7.50	0.76
(331) SB	triangular	3	7.83	7.50	0.75
(331) SE	triangular	3	5.67	5.50	0.40
(221) SB	triangular	4	8.00	7.83	0.78
(221) SE	triangular	4	5.67	5.50	0.32
(553) SB	triangular	5	7.83	7.83	0.82
(553) SE	triangular	5	5.67	5.50	0.32
100 TC @ Pt_{201}	-	-	-	6.33	0.61
100 SE @ Pt_{201}	-	-	-	5.17	0.28
111 SE @ Pt_{201}	-	-	-	5.00	0.23
kink @ Pt_{201}	-	-	-	4.25	0.11
Pt_{368} CD	-	-	-	7.83	0.80
Pt_{368} SE	-	-	-	5.17	0.41
Pt_{368} kink	-	-	-	4.08	0.04
Pt_{378}	-	-	-	7.83	0.83
Pt_{414}	-	-	-	7.67	0.77
Pt_{414}	-	-	-	7.83	0.85

S3. Experimental assessment of *OH adsorption energies

Experimentally, the apparent difference in the adsorption energies of certain adsorbates can be assessed using data obtained using voltammetry experiments [16,17]. This is possible when the interpretation of a peak is clear, which is the case for *OH on Pt at potentials in the range 0.6-0.8 V_{RHE}. First of all, adsorption isotherms are constructed by integrating the relevant part of the voltammogram, giving rise to a plot correlating *OH coverage and electrode potential (black curves in Figure S4). The differences in *OH adsorption energies, which are identical to the differences in *OH adsorption potentials, are determined at a coverage of 0.5 ML (blue lines in Figure S4).

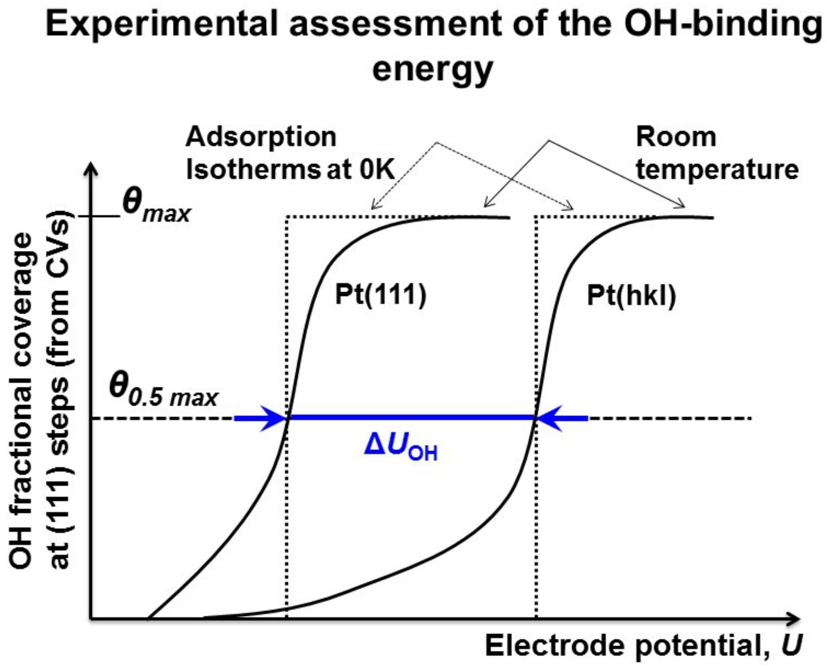

Figure S4. Schematics of the procedure used to estimate the relative change in *OH energy based on experimental voltammograms (in this case using integrated anodic parts of them). The difference between the potentials where half of the maximal fractional coverage is reached at different surfaces are considered to be a good approximation of the ΔU_{OH} between them.

S4. Electrochemical measurements

All original electrochemical experiments in this work were performed in a three-electrode electrochemical cell under so-called hanging meniscus configuration, as schematically shown in Figure S5.

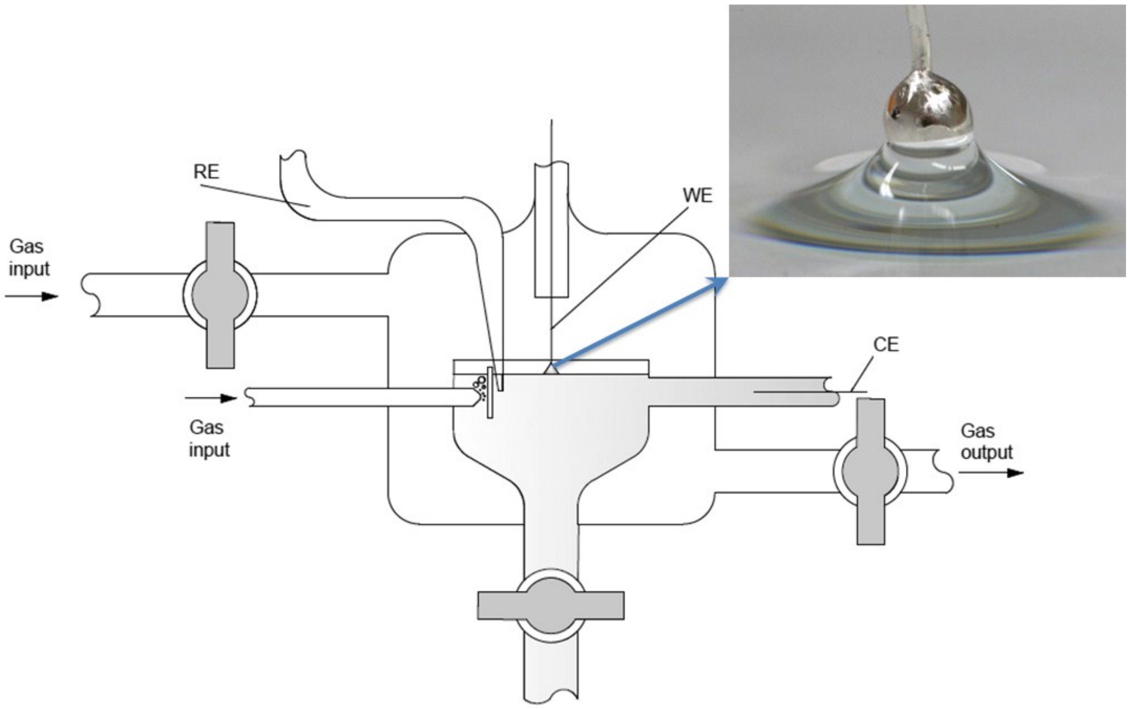

Figure S5. Schematics of the electrochemical setup used for in our experiments. WE: working electrode, RE: reference electrode, CE: counterelectrode.

Prior to the experiments, all glassware was cleaned with a 3:1 mixture of H_2SO_4 and H_2O_2 (both Suprapur, Merck, Germany) and consequently rinsed multiple times with ultrapure water from an Evoqua Ultra Clear 10 TWF 30 UV (Evoqua, Germany) water purification system. For the electrochemical measurements a VSP-300 potentiostat (Bio-Logic, France) was used. The working electrode was introduced into the electrolytes under potential control at 0.05V vs RHE prior to the measurement. Subsequently, the cyclic voltammograms were measured with a scan rate of 50 mV s^{-1} in Ar-saturated solutions (Ar 5.0, Air Liquide, Germany). As reference and counter electrodes, we used a mercury–mercury sulfate electrode (MMS) (SI Analytics, Germany) and a polycrystalline Pt wire, respectively. All measured potentials in this paper are presented in the RHE scale. The working electrolytes were prepared using 70% $HClO_4$ (Suprapur, Merck, Germany), by diluting it with ultrapure water.

S5. Generalized coordination numbers for some other electrocatalytic systems

Figure S6 shows the schematics of the initial stages of the coalescence of two Pt nanoparticles, in which sites with $\overline{CN} > 7.5$ are present near the junction.

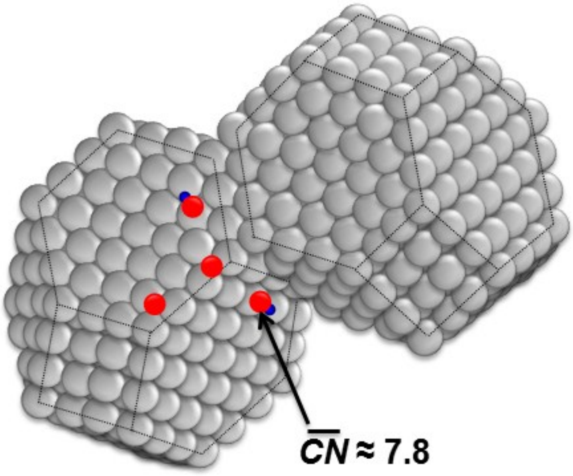

Figure S6. Schematic representation of the initial stage of coalescence of two convex nanoparticles. This situation is probable at high nanoparticle loading [18] and when nanoparticles form super-ordered arrays [19]. The arrow schematically shows an OH-adsorption site with the generalized coordination number greater than 7.5.

Figure S7 contains schematics of the mesoporous Pt ORR catalysts reported in [20].

(A) (B)

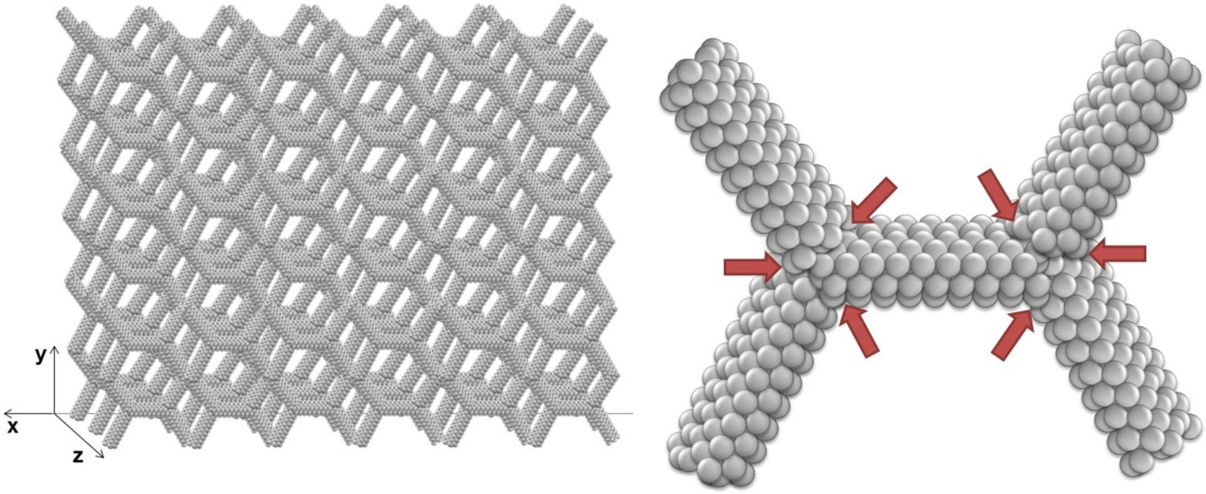

Figure S7. (A) Schematic representation of a porous Pt ORR electrocatalyst. (B) The arrows point to the surface sites where the generalized coordination number is larger than 7.5. The existence of these areas can explain the unexpectedly high (~1.5 times larger than that of Pt(111)) ORR specific activities of mesostructured Pt films reported in [20].

Figure S8 shows a comparison between pristine and missing-row-reconstructed Pt(110). Purple arrows indicate the location of the missing rows. Finally, Figure S9 shows all of the inequivalent sites on Pt_{201}, and 3 sites on Pt_{368} the activities of which appear in Figure 3 in the main text and in Table S3 in this Supporting Information.

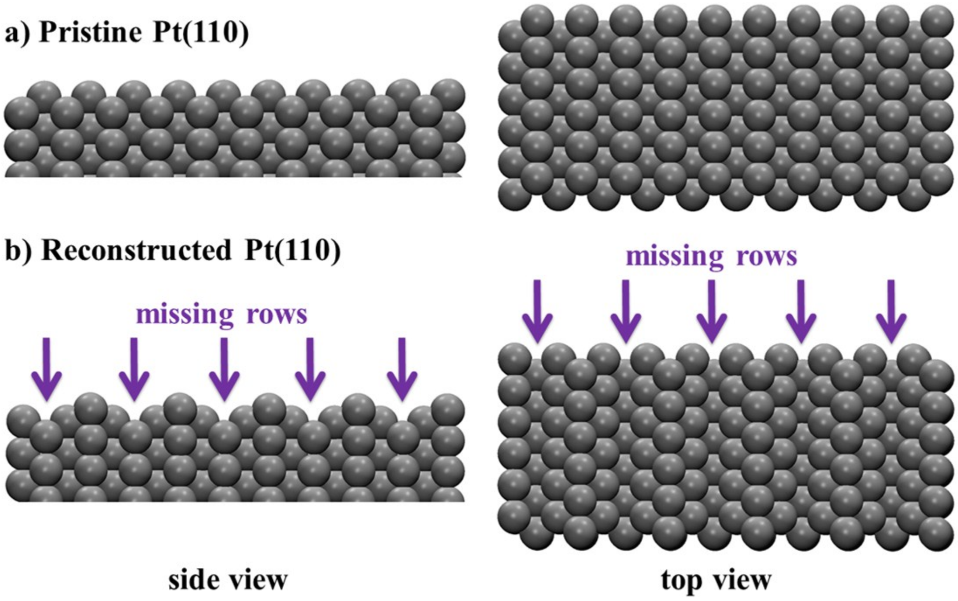

Figure S8. Side (left) and top (right) schematic views of Pt(110). a) Pristine, b) missing-row reconstructed.

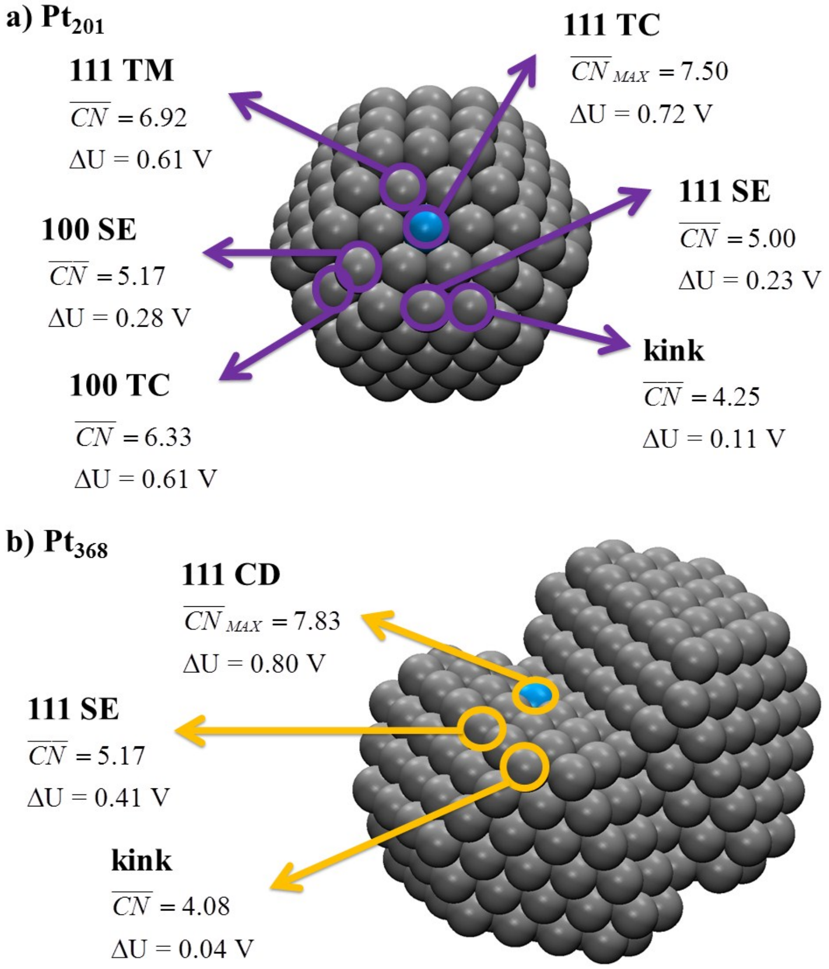

Figure S9. Surface active sites on Pt_{201}, a truncated-octahedron nanoparticle and on Pt_{368}, a concave nanoparticle. $\overline{CN}$ and the potentials (ΔU) required for the limiting steps to be thermodynamically favorable are provided for each site. TC: terrace center; TM: terrace middle; SE: step edge; CD: concave defect.

S6. Concave region of the coordination-activity plot

In Figure S10 we present a zoom in the concave region of Figure 3 in the main text to facilitate its detailed visualization.

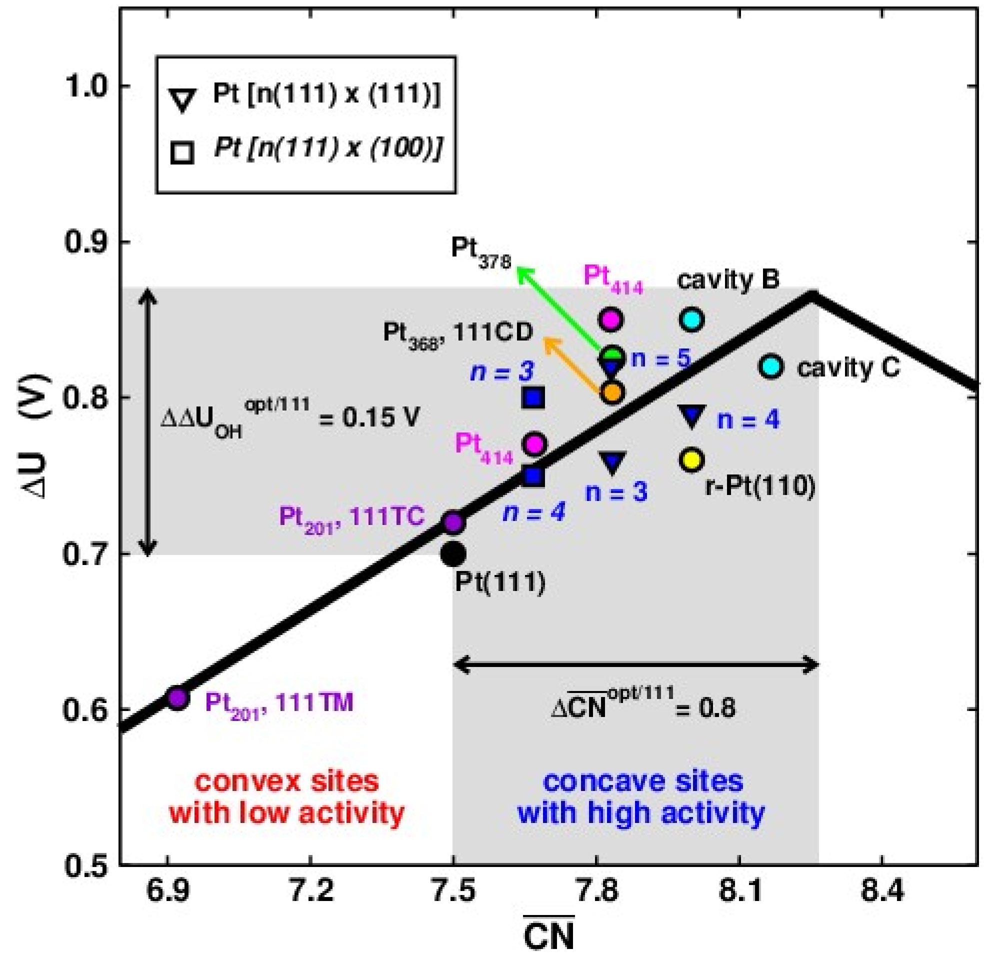

Figure S10. Concave region of the coordination-activity plot for the electrocatalysis of the oxygen reduction reaction on pure Pt sites.

References

1. Stamenkovic, V.R.; Fowler, B.; Mun, B. S.; Wang, G. F.; Ross, P. N.; Lucas, C. A.; Markovic, N. M. *Science* **2007**, *315*, 493.

2. Hitotsuyanagi, A.; Nakamura, M.; Hoshi, N. *Electrochim. Acta* **2012**, *82*, 512.

3. Kuzume, A.; Herrero, E.; Feliu, J. M. *J. Electroanal. Chem.* **2007**, *599*, 333.

4. Nørskov, J. K.; Rossmeisl, J.; Logadottir, A.; Lindqvist, L.; Kitchin, J. R.; Bligaard, T.; Jónsson, H. *J. Phys. Chem. B* **2004**, *108*, 17886.

5. Greeley, J.; Stephens, I. E. L.; Bondarenko, A. S.; Johansson, T. P.; Hansen, H. A.; Jaramillo, T. F.; Rossmeisl, J.; Chorkendorff, I.; Nørskov, J. K. *Nat. Chem.* **2009**, *1*, 552.

6. Calle-Vallejo, F.; Tymoczko, J.; Colic, V.; Vu, Q. H.; Pohl, M. D.; Morgenstern K.; Loffreda, D.; Sautet, P.; Schuhmann, W.; Bandarenka, A. S. *Science* **2015**, *350*, 185.

7. Stephens, I. E. L.; Bondarenko, A. S.; Grønbjerg, U.; Rossmeisl, J.; Chorkendorff, I. *Energy Environ. Sci.* **2012**, *5*, 6744-6762.

8. Calle-Vallejo, F.; Koper, M.T.M., *Electrochim. Acta* **2012**, *84*, 3.

9. Berna, A.; Climent, V.; Feliu J. M. *Electrochem. Commun*. **2007**, *9*, 2789.

10. Hansen, H. A.; Rossmeisl, J.; Nørskov, J. K. *Phys. Chem. Chem. Phys*. **2008**, *10*, 3722.

11. Bondarenko, A. S.; Stephens, I. E. L.; Hansen, H. A.; Perez-Alonso, F. J.; Tripkovic, V.; Johansson, T. P.; Rossmeisl, J.; Nørskov, J. K.; Chorkendorff, I. *Langmuir* **2011**, *27*, 2058.

12. Tripkovic, V.; Skúlason, E.; Siahrostami, S.; Nørskov, J. K.; Rossmeisl, J. *Electrochim. Acta* **2010**, 55, 7975.

13. Casalongue, H.S.; Kaya, S.; Viswanathan, V.; Miller, D. J.; Friebel, D.; Hansen, H. A.; Nørskov, J. K.; Nilsson, A.; Ogasawara, H. *Nat. Commun*. **2013**, *4*, 2817.

14. Calle-Vallejo, F.; Martínez, J. I.; García-Lastra, J.M.; Sautet, P.; Loffreda, D. *Angew. Chem. Int. Ed*. **2014**, *53*, 8253.

15. Peng, G.; Mavrikakis, M. *Nano Lett*. **2015**, *15*, 629.

16. Bandarenka, A. S.; Hansen, H. A.; Rossmeisl, J.; Stephens, I. E. L. *Phys. Chem. Chem. Phys*. **2014**, *16*, 13625.

17. Rossmeisl, J.; Karlberg, G. S.; Jaramillo, T.; Nørskov, J. K. *Faraday Discuss.* **2008**, *140*, 337.

18. Nesselberger, M.; Roefzaad, M.; Fayçal Hamou, R.; Biedermann, P. U.; Schweinberger, F. F.; Kunz, S.; Schloegl, K.; Wiberg, G. K. H.; Ashton, S.; Heiz, U.; Mayrhofer, K. J. J.; Arenz, M. *Nat. Mater.* **2013**, *12*, 919.

19. Kang, Y.; Ye, X.; Chen, J.; Cai, Y.; Diaz, R. E.; Adzic, R. R.; Stach, E. A.; Murray, C. B. *J. Am. Chem. Soc.* **2013**, *135*, 42.

20. Kibsgaard, J.; Gorlin, Y.; Jaramillo, T. F. *J. Am. Chem. Soc*. **2012**, *134*, 7758.
